# Food Supply and Seawater *p*CO_2_ Impact Calcification and Internal Shell Dissolution in the Blue Mussel *Mytilus edulis*

**DOI:** 10.1371/journal.pone.0024223

**Published:** 2011-09-16

**Authors:** Frank Melzner, Paul Stange, Katja Trübenbach, Jörn Thomsen, Isabel Casties, Ulrike Panknin, Stanislav N. Gorb, Magdalena A. Gutowska

**Affiliations:** 1 Biological Oceanography, Leibniz-Institute of Marine Sciences (IFM-GEOMAR), Kiel, Germany; 2 Laboratorio Maritimo da Guia, Faculdade de Ciencias da Universidade de Lisboa, Cascais, Portugal; 3 Institute of Zoology, Functional Morphology and Biomechanics, Christian-Albrechts-University, Kiel, Germany; 4 Institute of Physiology, Christian-Albrechts-University, Kiel, Germany; Heriot-Watt University, United Kingdom

## Abstract

Progressive ocean acidification due to anthropogenic CO_2_ emissions will alter marine ecosytem processes. Calcifying organisms might be particularly vulnerable to these alterations in the speciation of the marine carbonate system. While previous research efforts have mainly focused on external dissolution of shells in seawater under saturated with respect to calcium carbonate, the internal shell interface might be more vulnerable to acidification. In the case of the blue mussel *Mytilus edulis*, high body fluid *p*CO_2_ causes low pH and low carbonate concentrations in the extrapallial fluid, which is in direct contact with the inner shell surface. In order to test whether elevated seawater *p*CO_2_ impacts calcification and inner shell surface integrity we exposed Baltic *M. edulis* to four different seawater *p*CO_2_ (39, 142, 240, 405 Pa) and two food algae (310–350 cells mL^−1^ vs. 1600–2000 cells mL^−1^) concentrations for a period of seven weeks during winter (5°C). We found that low food algae concentrations and high *p*CO_2_ values each significantly decreased shell length growth. Internal shell surface corrosion of nacreous ( = aragonite) layers was documented via stereomicroscopy and SEM at the two highest *p*CO_2_ treatments in the high food group, while it was found in all treatments in the low food group. Both factors, food and *p*CO_2_, significantly influenced the magnitude of inner shell surface dissolution. Our findings illustrate for the first time that integrity of inner shell surfaces is tightly coupled to the animals' energy budget under conditions of CO_2_ stress. It is likely that under food limited conditions, energy is allocated to more vital processes (e.g. somatic mass maintenance) instead of shell conservation. It is evident from our results that mussels exert significant biological control over the structural integrity of their inner shell surfaces.

## Introduction

Progressive ocean acidification due to anthropogenic CO_2_ emissions will impact marine ecosytems [1]–[4]. Calcifying organisms may be particularly vulnerable to ocean acidification, as elevated seawater *p*CO_2_ shifts the carbonate system speciation towards a decreased concentration of carbonate ions. This leads to a reduced CaCO_3_ saturation state for aragonite and calcite (Ω_arag_ or Ω_calc_), which can negatively impact calcification rates in several marine heterotrophic taxa [2], [3]. Bivalve molluscs, particularly their early life stages, can react with decreased rates of growth and calcification, as well as decreased shell strength towards elevated seawater *p*CO_2_ [5]–[9]. Changes in growth and calcification performance encountered in bivalve molluscs most likely are related to an altered energy budget allocation, with more energy potentially being consumed by homeostatic processes [10], [11]. Exoskeleton dissolution can be observed in some gastropod and bivalve mollusk species when Ω drops ≪1 [12]–[14]. Species vulnerability to external shell dissolution is probably strongly related to the presence or absence of a protective organic cover on the external shell side [15], [16] and, potentially, the protein and carbohydrate organic matrix that surrounds CaCO_3_ crystals within the shell [17]–[19]. While Ω<1 is rare in the contemporary global surface ocean [20], low Ω is a common feature of temperate coastal habitats, where seasonal hypoxia goes along with dissolved inorganic carbon production. Wind driven upwelling can then bring CO_2_ enriched waters in contact with shallow water habitats [15], [21]. We have recently measured very high and fluctuating summer seawater *p*CO_2_ (>100 Pa, >1000 µatm, Ω_arag_≪1) in habitats in the Western Baltic that are dominated by the blue mussel *Mytilus edulis*. We could also demonstrate that mussels from this population can calcify at high rates even when Ω_arag_<0.5 when food supply is abundant [10], [15]. While high calcification rates in seawater under saturated with CaCO_3_ already seem remarkable, it needs to be emphasized that the extracellular environment at the inner shell interface is even less favorable for biomineralization: as all heterotrophic marine ectothermic animals maintain *p*CO_2_ values in their extracellular fluids (i.e. hemolymph, extrapallial fluid) between 100–400 Pa (ca. 1000–4000 µatm) in order to drive diffusive excretion of metabolic CO_2_ [22], the extracellular carbonate system is shifted further towards decreased [CO_3_^2−^]. While it cannot be excluded that the precise site of incipient biomineralization is occluded by an organic matrix sheath or gel to create a microenvironment that is characterized by higher Ω_arag_ [19], [23], [24], inner shell regions that are not actively being expanded are in contact with an extracellular fluid that is most likely highly corrosive (low pH and [CO_3_^2−^], high *p*CO_2_). Some protection from dissolution may be provided by the chitin and protein layer that covers the uppermost (mantle facing) nacre [18]. During exposure to high seawater *p*CO_2_, the extracellular carbonate system shifts to even lower carbonate concentrations, as *M. edulis* and other bivalves do not perform an extracellular pH (pHe) compensatory reaction [11], [15]. While these results indicate strong biological control over the biomineralization process in mussels, they also suggest that a continuous energetic effort may be necessary to maintain inner shell integrity. It has been previously shown that during stress (e.g. aerial exposure, environmental anoxia), the inner shell surface is corroded in several bivalve species due to proton generation through anaerobic production of succinate [25], [26]. While this may be adaptive, as CaCO_3_ helps to buffer the developing acidosis, it also indicates that the inner shell might be more endangered by elevated seawater *p*CO_2_ than the outer shell surface, which is covered by a chemically resistant periostracum [27]. The situation may be different in those calcifiers (e.g. cephalopods, teleost fish, decapod crustaceans) that actively modulate the extracellular carbonate system speciation in order to stabilize pHe: these organisms accumulate [HCO_3_^−^]e significantly above seawater levels (ca. 2 mM). This leads to high calcium carbonate saturation states and could be one reason for the observed occurrence of increased rates of calcification or ‘hypercalcified’ skeletal structures [3], [28]–[30].

We predict that when elevated seawater *p*CO_2_ (hypercapnia) is coupled with food limitation, the inner shell front may be the area of the bivalve shell that is most endangered to suffer from partial dissolution or corrosion. Recent studies indicate that CO_2_ effects on biomineralization in several marine heterotrophic calcifiers that are weak acid-base regulators are primarily due to altered priorities in energy budget allocation [10], [31], [32]. However, studies that tested the influence of altered energy supply on energy budget decisions during CO_2_ stress are lacking to date. To systematically address this research question, we performed a seven – week long experiment in which we monitored the effects of food algae concentration (2 levels) and *p*CO_2_ (4 levels) on *M. edulis* shell growth and shell integrity. Following the experiment, we investigated inner shell surfaces using stereo microscopic and SEM techniques.

## Materials and Methods

### Animals and experimental incubation

Mussels were collected on 2010-02-22 from a subtidal population in Kiel Fjord (54°19.8′N; 10°9.0′E) and directly transferred to the experimental aquaria. We consider Kiel Fjord blue mussels *M. edulis*, although introgression of *M. trossulus* alleles has recently been observed for some nuclear markers in our experimental population [33]. Once in the experimental aquaria, *p*CO_2_ was adjusted within 5 hours to the target values. The experiment lasted from 2010-02-22 until 2010-04-08. Each aquarium contained 4 experimental animals (see Table 1 for details). Experimental design was essentially identical to that presented in Thomsen & Melzner [10], except that in this experiment we used 32 aquaria, of which 16 were assigned to the high food density group (HF) and 16 to the low food density group (LF). Each 4 experimental aquarium units were continuously equilibrated at each food level with pre-mixed gases containing 390 ppmv, 1120 ppmv, 2400 ppmv, 4000 ppmv CO_2_ using a central automatic CO_2_ mixing-facility (Linde Gas & HTK Hamburg, Germany). The CO_2_ treatment levels were chosen to represent conditions that can be expected to seasonally occur in Kiel Fjord within the next 100 years (see [15] for rationale). Experimental aquaria (ca 18 liters each) were continuously perfused with (50, 20, 5 µm) filtered and UV treated seawater from the fjord at a rate of 50 mL min^−1^ via gravity feed from two header tanks. Flow rates to the experimental aquaria were checked and adjusted daily. Food algae (*Rhodomonas* sp.) were cultured as previously described [15] and supplemented continuously to the 16 HF tanks to maintain a cell density of 1600–2000 cells mL^−1^ in the experimental units (see Table 1 for details). The 16 LF tanks received no additional food in addition to what passed though the filters and cell density was maintained at 310–350 cells mL^−1^ (see Table 1). Food algae density was measured on four occasions during the experimental incubation using a Coulter counter (cells between 4–10 µm diameter). Salinity, temperature and pH (NBS scale) were measured daily using a WTW 340i pH-meter and a WTW SenTix 81-electrode which was calibrated with Radiometer IUPAC precision pH buffer 7 and 10 (S11M44, S11 M007), the light∶dark cycle was adjusted to 12∶12 hours. Carbonate chemistry of the seawater was determined twice during the experimental period (week 2, week 7) from pH_NBS_ and by measuring total dissolved inorganic carbon (*C*_T_) using an AIRICA autoanalyzer (Marianda GmbH, Kiel, Germany) with a precision of 2–4 µmol kg^−1^ seawater. Accuracy of *C*_T_ measurements was ensured by using Certified Reference Material provided by Andrew Dickson of the Scripps Institution of Oceanography (http://andrew.ucsd.edu/co2qc/). Seawater carbonate system parameters (Ω, *p*CO_2_) were calculated using the CO2SYS program [34]. The carbonate system speciation (*p*CO_2_, Ω_calc_ and Ω_arag_) was calculated from pH and alkalinity using CO2SYS ([22], Table 1) with dissociation constants from Roy et al. [35].

**Table 1:** Water parameters and mussel growth.

A											
F1	F2	cell density header (cells mL^−1^)		cell density aquaria (cells mL−1)		pH_NBS_		T (°C)		S	
HF	39	5688	(4200)	1655	(242)	8.01	(0.04)	4.8	(0.7)	16.0	(1.3)
HF	112			1575	(55)	7.69	(0.04)	4.7	(0.7)	16.0	(1.3)
HF	240			1747	(323)	7.35	(0.05)	4.8	(0.7)	16.0	(1.3)
HF	405			1962	(166)	7.15	(0.04)	4.9	(0.7)	16.0	(1.4)
LF	39	575	(307)	314	(63)	8.01	(0.04)	5.4	(0.6)	16.0	(1.3)
LF	112			335	(13)	7.70	(0.04)	5.1	(0.5)	16.0	(1.3)
LF	240			347	(25)	7.40	(0.04)	5.0	(0.6)	16.0	(1.3)
LF	405			346	(18)	7.19	(0.04)	4.9	(0.5)	16.0	(1.3)

B													
F1	F2	*C*_T_ (µmol kg^−1^)		pH_NBS_		*A*_T_ (µmol kg^−1^)		*p*CO_2_ (Pa)		Ω_calc_		Ω_arag_	
HF	39	1881.9	(13.7)	8.05	(0.01)	1915.2	(13.8)	53	(2)	1.23	(0.05)	0.72	(0.03
HF	112	1951.8	(11.0)	7.72	(0.03)	1914.7	(10.2)	119	(8)	0.58	(0.04)	0.34	(0.02)
HF	240	2033.4	(9.4)	7.37	(0.02)	1897.8	(10.5)	262	(14	0.26	(0.02)	0.15	(0.01)
HF	405	2097.3	(13.3)	7.19	(0.03)	1880.9	(10.3)	396	(23)	0.17	(0.01)	0.10	(0.01)
LF	39	1899.6	(38.9)	8.05	(0.04)	1933.8	(36.8)	55	(5)	1.26	(0.13	0.73	(0.07)
LF	112	1929.0	(3.5)	7.73	(0.04)	1896.3	(9.2)	114	(10)	0.61	(0.08)	0.35	(0.05)
LF	240	2015.1	(7.3)	7.43	(0.05)	1898.3	(17.7)	234	(27)	0.30	(0.05)	0.18	(0.03)
LF	405	2084.5	(11.1)	7.18	(0.03)	1862.4	(17.3)	401	(28)	0.17	(0.01)	0.10	(0.01)

C											
F1	F2	initial shell length (mm)		length growth (mm)		shell mass growth (mg)		somatic mass growth (mg DM)		nacre dissolution (% shell area)	
HF	39	16.8	(1.4)	2.6	(1.2)	70.4	(61.9)	34.7	(27.2)	0.0	(0.0)
HF	112	17.6	(1.1)	3.0	(0.8)	72.6	(53.0)	40.7	(30.5)	0.3	(0.2)
HF	240	16.8	(0.7)	2.5	(0.4)	39.9	(20.1)	29.8	(14.8)	4.0	(5.3)
HF	405	16.7	(0.5)	1.8	(0.5)	44.2	(18.8)	36.5	(12.1)	13.5	(5.2)
LF	39	16.6	(0.5)	1.2	(0.3)	24.3	(4.8)	5.0	(6.1)	3.8	(6.7)
LF	112	17.3	(0.9)	1.5	(0.4)	21.8	(11.2)	7.0	(9.4)	3.9	(3.8)
LF	240	17.4	(1.0)	1.1	(0.3)	39.5	(13.5)	17.4	(16.1)	8.2	(3.7)
LF	405	16.7	(0.9)	0.6	(0.2)	8.9	(8.2)	8.5	(17.2)	34.1	(6.9)

(A) seawater parameters during the experimental incubation, daily measurements (T, S, pH) and averages of four determinations (cell densities in aquaria and header tanks); (B) carbonate system speciation calculated from pH_NBS_ and total dissolved inorganic carbon (*C*_T_) measurements, mean values of two determinations in all 32 experimental tanks; (C) mussel growth trial results. Means and standard deviations (in brackets). F1 = factor 1, food level; F2 = factor 2, *p*CO_2_, *A*_T_ = total alkalinity. A seawater *p*CO_2_ of 100 Pascal (Pa) corresponds to 987 µatm.

Mussels were sacrificed after an incubation time of 7 weeks. Shell length was determined using a caliper (precision 0.1 mm). Mussels were carefully opened with a scalpel and the soft body was dissected without injury of the inner shell layers. Somatic tissue was dried at 80°C for 24 h and weighed on a precision scale (Sartorius TE64, Sartorius AG, Germany). Shell dry mass was determined following 24 h of drying at 60°C. Shell length growth was directly obtained from initial and final length measurements, while shell mass growth and somatic growth were estimated from the difference between final measurements and estimates of initial shell- and somatic mass, obtained using a shell length vs. somatic mass and a shell length vs. shell mass relationships from a subsample of mussels (N = 10 mussels, shell length range: 13–23 mm) sampled on 2010-02-22 from the pool of animals intended for this experiment:
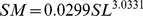
(1)where SM = shell mass in mg, SL = shell length in mm, R^2^ = 0.98, p<0.01.
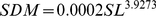
(2)where SDM = somatic dry mass in mg, SL = shell length in mm, R^2^ = 0.95, p<0.01.

### Shell analysis

One valve from each experimental mussel (i.e. 4 shells per replicate aquarium) was analyzed under the stereomicroscope (Wild Heerbrugg, Leica Microsystems, Wetzlar, Germany) at 8–40× magnification and images were taken using a digital camera (ProgRes CF, Jenoptik, Jena, Germany) and ProgRes Picture Pro 2.7 software (Jenoptik) at 10× magnification. Most shells from the higher *p*CO_2_ treatments were characterized by partial corrosion of the nacre, the innermost shell layer. Corroded regions lose the typical glossy appearance of nacre and appear white when viewed under a light source (see Fig. 2a). We thus were able to distinguish between corroded parts of the shell and intact regions using the freeware ImageJ Version 1.43 (http://rsbweb.nih.gov/ij/) based on differences in grey scale. Grey scale thresholds were adjusted for each valve based on grey scale intensity differences of visually confirmed corroded vs. uncorroded areas of the particular shell image (uncorroded nacre reflects visible light). Corroded areas were marked and set in relation to the total inner surface area of the valve:
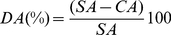
(3)where DA(%) = dissolved nacre area in % of the total inner shell area, SA = inner shell area, i.e. all pixels enclosed by the shell circumference, CA = corroded area, i.e. nacre pixels above a set grey scale threshold. The curvature of the shell was ignored for area calculations. All shells were similar in phenotype; total shell growth (1–3 mm) during the experimental incubation was small enough relative to shell length at the end of the experiment (18–21 mm) in order to neglect potential differences in shell curvature between treatments.

SEM images were generated from select shells to confirm our classification into control and corroded nacre regions. Valves were broken along defined trajectories and 2 cm long shell fractions were mounted on SEM pedestal stubs. Sections were coated with gold-palladium and examined using scanning electron microscopes (Nanolab 7, Zeiss, Oberkochen, Germany and Hitachi S4800, Hitachi High - Technologies Europe, Krefeld, Germany).

### Viscosity and filtration calculations

Seawater kinematic viscosity (ν, 10^−6^ m^2^ s^−1^) was calculated for experimental conditions (mean temperature and salinity) for this and two previous experiments [10], [15]. Viscosity in relation to temperature was calculated (>10°C) and extrapolated (<10°C) from values published by El-Dessouky and Ettouny [36]. Filtration rates (FR) of 9.6 ml min^−1^ were calculated for a standard mussel of 18 mm shell length and 29.1 mg dry mass at 15°C and a corresponding viscosity of 1.15 10^−6^ m^2^ s^−1^ according to Riisgård and Møhlenberg [37]. The obtained rate resembles FR for mussel sizes used in our experiments. Riisgård and Larsen [38] described a linear decrease of FR with increasing viscosity. Assuming a decrease of 10% per 0.1 10^−6^ m^2^ s^−1^ viscosity increase (calculated from experimental data in [38] and references therein), FR was recalculated for the mean viscosity in the experimental aquaria.

### Statistics

Measurements from the four mussels of each replicate aquarium were averaged. Two-factorial ANOVA was used to analyze shell length, mass growth, somatic mass growth and internal shell corrosion in response to food supply (2 levels, 4 replicate aquaria each) and seawater *p*CO_2_ (4 levels, 4 replicate aquaria each) using Statistica 8. Relative quantities (i.e. % shell corrosion area) were arcsine transformed prior to statistical analysis.

## Results

**Figure 1:**
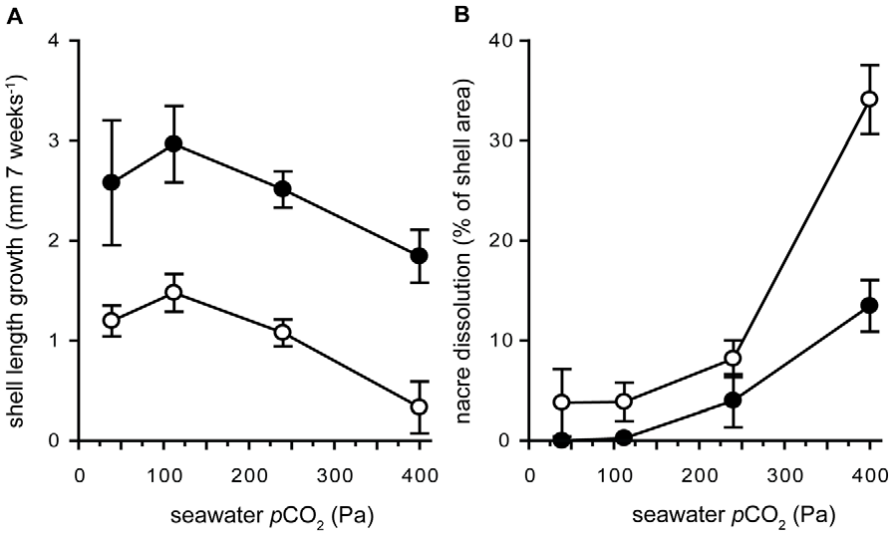
Shell length growth (A) and internal shell surface dissolution (B) vs. seawater *p*CO_2_. (A) Shell length growth during the seven week growth trial. (B) Internal shell surface dissolution in % of total inner shell area; low food (LF, white) and high food (HF, black) groups, means and SEM. A seawater *p*CO_2_ of 100 Pa = 987 µatm.

Following seven weeks of acclimation to 2 food and 4 CO_2_ treatment levels, experimental animals were sampled. Shell length growth was significantly reduced by low algae cell density and by high seawater *p*CO_2_, with no significant interaction between factors (Fig. 1, Table 1, 2). With respect to controls at each food level, shell length growth was significantly reduced in the highest *p*CO_2_ treatment (405 Pa, p<0.001). Shell mass growth and somatic growth were significantly reduced in the LF treatment (Table 2). However, no significant differences in shell mass and somatic growth were found with respect to seawater *p*CO_2_. It needs to be mentioned that absolute rates of growth were low in this experiment in comparison to previous experiments at higher temperatures (see Table 3). While shell length growth was based on direct initial and final measurements, initial shell and somatic mass were interpolated (see Methods). Owing to large variability of shell length vs. shell mass relationships (see e.g. [15]), shell and somatic growth rates estimated in this experiment should be viewed with caution.

**Table 2:** Two - factorial ANOVA results. Significant results in bold.

A length growth					
	SS	d.f.	MS	F	p
intercept	101.7	1	101.7	281.4	<0.0001
***p*** **CO_2_**	**4.1**	**3**	**1.4**	**3.8**	**<0.03**
**food**	**15.4**	**1**	**15.4**	**42.5**	**<0.0001**
*p*CO_2_*food	0.1	3	0.02	0.1	<0.98
error	8.7	24	0.4		

B shell mass growth					
	SS	d.f.	MS	F	p
intercept	49083.3	1	49083.3	49.1	<0.0001
*p*CO_2_	3304.9	3	1101.6	1.1	<0.37
**food**	**9922**	**1**	**9922**	**9.9**	**<0.005**
*p*CO_2_*food	3288.4	3	1096.14	1.1	<0.37
error	23980.4	24	999.2		

C shell dissolution					
	SS	d.f.	MS	F	p
intercept	1.582	1	2	152	<0.0001
***p*** **CO_2_**	**0.903**	**3**	**0.301**	**29**	**<0.0001**
**food**	**0.209**	**1**	**0.209**	**20**	**<0.0002**
*p*CO_2_*food	0.021	3	0.007	0.69	<0.57
error	0.250	24	22		

D somatic growth					
	SS	d.f.	MS	F	p
intercept	16141.3	1	16141.3	47.5	<0.0001
*p*CO_2_	81.5	3	27.2	0.1	<0.98
**food**	**5389.2**	**1**	**5389.2**	**15.9**	**<0.0006**
*p*CO_2_*food	522.7	3	174.2	0.5	<0.68
error	8157.8	24	339.9		

**Table 3:** Comparison of control *p*CO_2_ treatments of *M. edulis* growth trials conducted in our laboratory.

Experiment	S	T	Ω_arag_	kinematic viscosity	calculated filtration rate	cell density(aquaria)	shell lengthgrowth
	g kg^−1^	°C	control	10^−6^ m^2^ s^−1^	ml min^−1^	n ml^−1^	% initial
Thomsen et al. (2010)summer 2009	15.0±0.6	13.8±0.6	1.14±0.04	1.19±0.019	9.2	820±315	64.8±3.8
Thomsen and Melzner (2010)autumn 2009	18.1±1.0	9.1±0.4	1.11±0.12	1.36±0.017	7.6	2330±482	53.0±2.0
this study HFwinter 2010	16.0±1.3	4.9±0.7	0.72±0.03	1.53±0.003	6.0	1734±167	15.2±7.3
this study LFwinter 2010	16.0±1.3	5.4±0.6	0.73±0.07	1.51±0.009	6.1	336±16	7.0±1.8

The summer and autumn experiments lasted 8 weeks [10], [15], the present winter experiment 7 weeks. Filtration rates were calculated for a standard 18 mm mussel (see methods) to illustrate the impact of temperature dependent changes in seawater viscosity. As *M. edulis* has a retention rate of ca. 100% for particles with a diameter >4 µm, reduced filtration rates at low temperatures directly translate into reduced energy uptake, even if aquarium algae concentrations are similar. See Methods for calculation of filtration rates in response to seawater viscosity. Means and standard deviations (in brackets). Shell length growth corresponds to growth during the entire experimental period of the respective experiments. Cell density refers to the average *Rhodomonas* cell density in the experimental aquaria. Ω_arag_ refers to the aragonite saturation states in the experimental aquaria.

Kinematic viscosity was ca. 30% higher at the low temperatures in this experiment than in our summer experiment (Table 3), this should have decreased filtration rates and food intake of the experimental mussels by 35%. This could partially explain lower shell growth rates observed in the present experiment under HF conditions: shell length growth during 8 week growth trials at warmer temperatures [10], [15] vs. that observed in the present 7 week growth trial was more than a factor three higher (Table 3).

**Figure 2:**
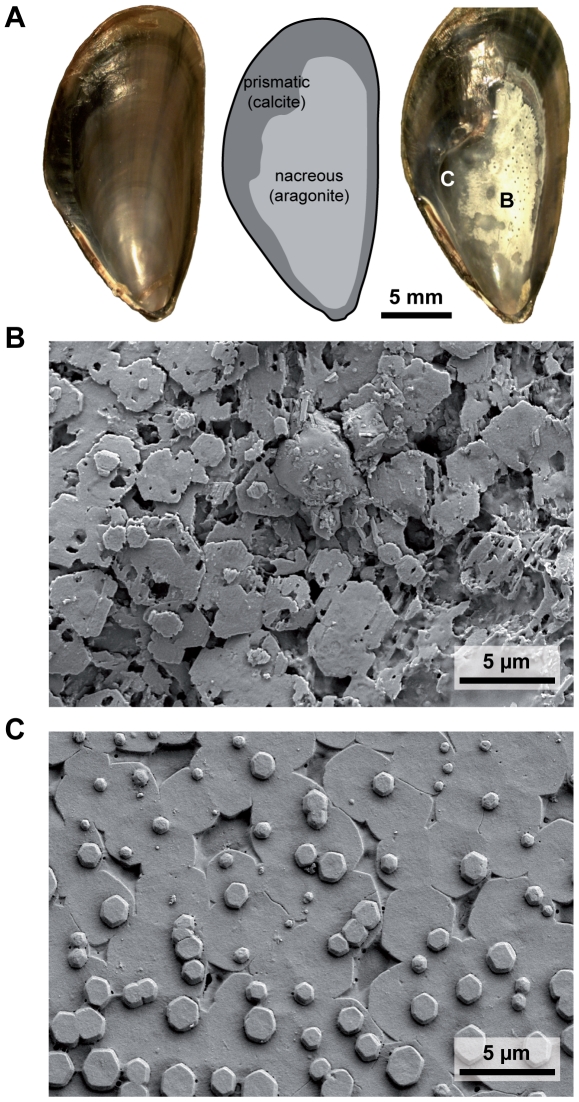
Internal shell dissolution of the nacreous layer. (A) left: stereo microscopic image of the inner shell surface of a control (39 Pa, HF) mussel, middle: schematic drawing of the inner shell surface area of the same control shell to indicate extent of the nacreous layer, right: inner shell surface of a high *p*CO_2_ (405 Pa, LF) mussel. White areas correspond to corroded nacreous surface layers, darker areas to unimpacted nacreous surfaces. (B) SEM image of a severely corroded nacre surface corresponding to position (B) in Fig. 2A. (C) SEM image of control nacre surfaces, illustrating coordinated aragonite tablet growth (3 layers visible).

**Figure 3:**
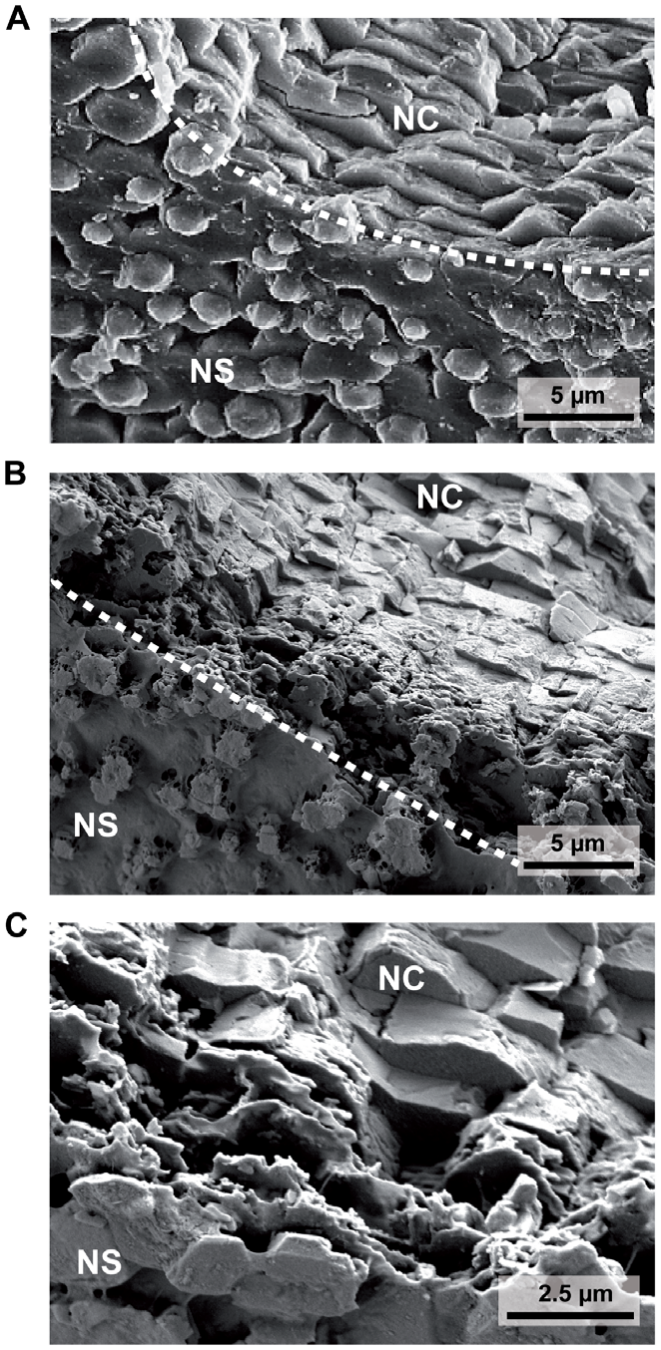
SEM images of shell cross sections of uncorroded (A) vs. corroded (B, C) nacreous tablet layers. (A) cross section through uncorroded nacreous layer with growing nacre tablets on the surface. (B, C) cross sections through dissolved nacreous layers, illustrating extensive dissolution of plates ca. 3–5 µm deep into the nacreous layer. Deeper nacreous layers appear to be macroscopically unaffected. Corroded cross sections were obtained from the same mussel piece shown in Fig. 4A and B. The white dashed lines in A, B indicate the breaking edge, NS = nacreous layer surface, NC = nacreous layer cross section.

Internal shell corrosion was visible in HF mussels only at the two highest CO_2_ treatment levels, while in the LF treatment, inner shell corrosion was observed in all four treatments (Fig. 2). The white color of corroded shell surfaces resulted from dissolution of nacre tablets and the resulting changes in light refraction due to the remaining organic material (Fig. 2). Shell cross sections illustrated that a visible dissolution zone extended ca. 3–5 µm into the nacreous layer (N = 6 shells studied, Fig. 3). Below the thin dissolution zone, nacre tablet layers appeared to be intact (Fig. 3b, c). The extent of inner shell surface corrosion was significantly influenced by both, *p*CO_2_ and food density (Fig. 1, Table 2). At the highest *p*CO_2_, all LF mussels were heavily corroded, with >30% of total inner shell area being affected.

**Figure 4:**
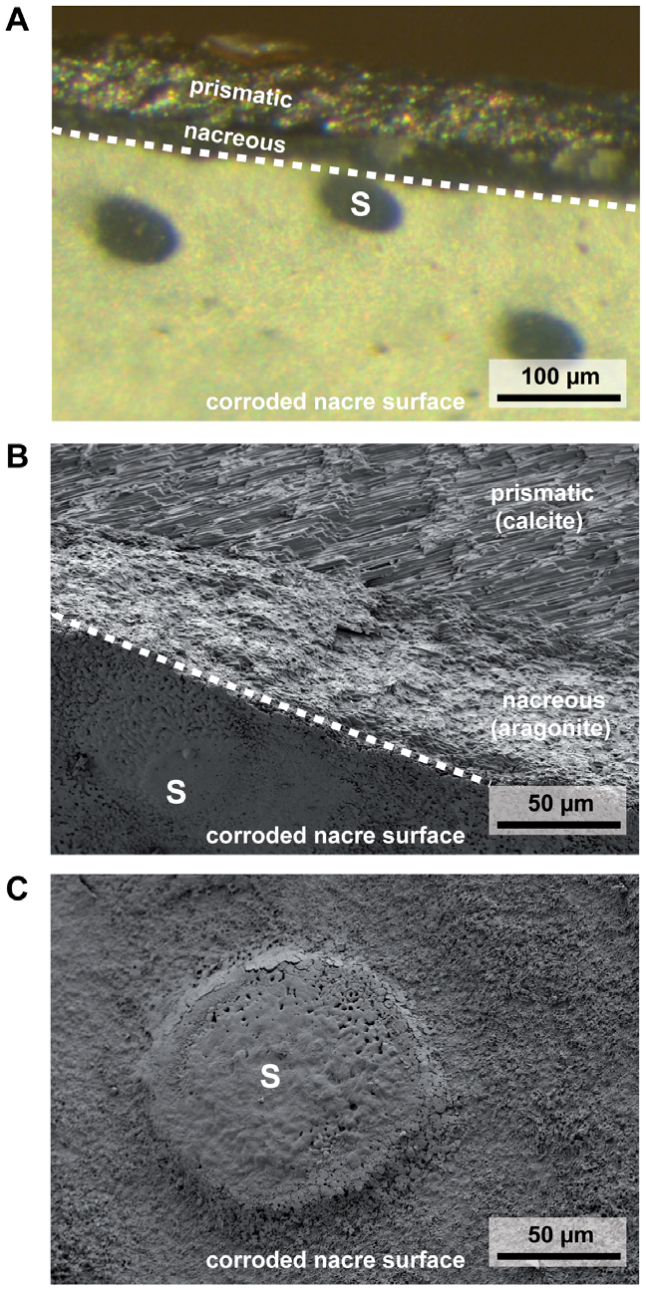
Stereo microscopic (A) and SEM (B,C) images of shell sections with organic covered spots on otherwise dissolved nacreous surfaces. (A) cross section through a shell piece with corroded nacre surface (white) and organic covered spots (blue), see Fig. 3 for larger SEM magnification of the upper part of the shell cross section. (B) SEM image of the same shell piece displayed in (A), illustrating nacreous and prismatic layers, as well as an amorphous structure on the location of the blue spot (S) which presumably is transparent and organic, as the characteristic light refraction pattern of the underlying nacreous layer is conserved in ‘spot’ areas, see (A). (C) SEM image from strongly corroded shell area, with blue spot elevated several µm above the surface. This shell region also appears to be covered with protective organic material.

Circular blue spots on the dissolved nacre surface appeared to be characteristic for heavily corroded shells (Fig. 4a). SEM revealed that these spots correspond to nacre regions that are covered by (transparent) organic material, which seems to protect the underlying nacre and its characteristic light refraction patterns (Fig. 4b,c). Fig. 4c illustrates an extreme case, where such a circular, organic covered spot extends several µm above the otherwise corroded nacre surface. Using SEM, multiple of such organic covered spots of 10–100 µm diameter could be demonstrated on N = 6 heavily corroded shells.

## Discussion

We acclimated blue mussels to four different seawater *p*CO_2_ values and two food concentrations at low temperatures and found effects of both factors on shell length growth. In addition, we were able to demonstrate internal shell corrosion in bivalves as a consequence of high seawater *p*CO_2_. We also found that internal shell corrosion is highly dependent on food concentration. Our results demonstrate the high degree of control that bivalves can exert over the structural integrity of the inner shell surface and suggest that shell corrosion is related to energy budget reallocations.

### Shell and somatic growth

While there was positive shell and somatic growth in all experimental groups during the experimental period, shell length and mass growth as well as somatic growth rates were lower than rates observed in previous summer and autumn experiments using experimental animals of comparative size from the same mussel population (Table 3) [10], [15]. While food supply was abundant in all three experiments, temperature was significantly lower (ca. 5°C) in the present experiment (Table 3). Temperature influences key processes that can impair growth and calcification in mytilid bivalves: (i) temperature induced reductions in metabolic rates are not fully compensated for by thermal acclimation of key physiological functions in mussels (e.g. cardiovascular function, [39]). In addition, (ii) seawater kinematic viscosity is strongly temperature dependent and was much higher in this experiment (by ca. 27%) than in the summer experiment (Table 3). Viscosity impacts mussel energy budgets as the lateral gill cilia that generate the feeding current are mechanically limited when working under conditions of high viscosity: Riisgård & Larsen [38] could demonstrate that temperature induced reductions in *M. edulis* filtration rate are exclusively due to increases in viscosity that go along with low temperature. We estimate that the mussels in this experiment filtered, on average, 34% less water than those in our summer experiment (Table 3) [15] As food retention of particles >4 µm is almost 100% [40], reduced filtration equates to reduced energy intake. Finally, (iii) CO_2_ solubility is higher in cold waters and CaCO_3_ saturation states are lower (see Table 1), which led to low Ω_arag_<1 in all treatments in the present experiment. This might also increase costs for calcification and shell maintenance. Low rates of shell length growth of <0.5 mm week^−1^ in winter animals (vs. >1 mm week^−1^ in summer) have also been detected *in situ* in Kiel Fjord mussels (see Fig. 2 in [15]). Food concentrations utilized in our experiments approximately correspond to Chl *a* values that can be expected in Kiel Fjord: assuming a Chl *a* content of 1–2 pg cell^−1^ *Rhodomonas* sp. [41], the HF treatment contained ca. 2–4 µg Chl *a* L^−1^ while the LF treatment contained 0.3–0.7 µg Chl *a* L^−1^. Kiel Fjord Chl *a* concentrations can be >10 µg L^−1^ during August and are typically below 1 µg L^−1^ in the winter months [42].

Shell length growth was significantly reduced in LF treatments and by high seawater *p*CO_2_, effects of both factors were more or less additive. No significant CO_2_ effects could be found with regard to shell mass and somatic mass growth, while food supply had a strong effect on both parameters. Variability of shell and somatic mass growth rate was high (Table 2, see methods), this might have potentially obscured more subtle CO_2_ effects. Similarly, other studies that recorded little total net shell and somatic growth also were unable to detect significant effects of *p*CO_2_ on calcification performance in juvenile/adult mytilid mussels at seawater *p*CO_2_ up to 300–500 Pa [3], [43]. Under elevated feeding conditions and at higher temperatures >9°C, strong reductions in shell length and mass accretion of >30% were found at high seawater *p*CO_2_ of >400–500 Pa, in both, brackish and fully marine mytilid populations [10], [15], [44].

### Internal shell dissolution

It has long been recognized that the inner bivalve shell surface is plastic and prone to dissolution. Dugal [45] was among the first to measure internal CaCO_3_ dissolution during emersion in the bivalve *Mercenaria mercenaria*, other studies followed by demonstrating that ^45^Ca that is incorporated into the shell appears in the extrapallial fluid during emersion [25], [46]. Akberali et al. [47] studied SEM images of inner shell surfaces of freshly exposed intertidal bivalves (*Scrobicularia plana*) and could demonstrate visible changes in the structural integrity of the inner shell layers during a single low tide event, leading the authors to conclude that dissolution and re-calcification are cyclic events on a daily basis. Further, these authors could also demonstrate that during abrupt 3 week - long exposure to dilute seawater (20%), shell mass decreased by 10% due to valve closure and subsequent internal dissolution. While Crenshaw & Neff [25] suggested that shell dissolution is primarily due to acidification caused by accumulation of the anaerobic endproduct succinate, Jokumsen & Fyhn [48] measured increases in extracellular [Ca^2+^] during aerial exposure in *M. edulis*, but also increases in extracellular *p*CO_2_, which they attributed to frequent brief valve opening to enhance residual aerobic metabolism. Thus, CO_2_ induced corrosion of the inner shell surface might be a common feature of mytilid physiology that is adaptive to life in intertidal habitats.

Long – term exposure to elevated *p*CO_2_ in our experiment induced shell corrosion of aragonite platelets that form the nacreous inner layer of the shell (Figure 2, 3, 4). This effect was more pronounced when mussels were food limited. It has been shown that bivalves do not control pHe in haemolymph and extrapallial fluid [15], [49]. Increases in seawater *p*CO_2_ lead to equivalent increases in extrapallial fluid and haemolymph *p*CO_2_ in order to maintain diffusion gradients to excrete metabolic CO_2_ (see [22] for a discussion). High *p*CO_2_ values of extracellular fluids constrain the carbonate system towards CO_3_^2−^ concentrations that are even lower than in the surrounding seawater (see Table 2), causing extremely low Ω_arag_ and low pH, thus progressively more corrosive conditions at the mantle shell interface. It is therefore likely that maintenance of inner shell integrity requires continuous energy investment. The strong effects of food availability and *p*CO_2_ (Table 2) point in this direction. Increased effort for inner shell maintenance might explain part of the surplus metabolism observed in bivalves exposed to elevated seawater *p*CO_2_ [5], [10], [11]. In highly corroded shells we also found regions that appeared to be covered with organic material (Fig. 3, 4). While these structures deserve further research attention with regards to their composition, it is intriguing to speculate that *M. edulis* may need to invest additional energy into synthesizing organic material in order to stabilize sites of dissolution. This is particularly relevant, as it has been suggested by Palmer [50] that the main costs of shell formation are those for organic matrix (i.e. the energetic value of the organic fraction within the shell and the biosynthesis costs for this material), with 29 J mg^−1^ shell in comparison to only 1–2 J mg^−1^ shell for CaCO_3_ deposition. Based on these results, an increase in the organic fraction of a shell from e.g. 1.5 to 5% would increase the relative costs for the organic fraction from 22% to almost 50% of shell formation costs (c.f. [50]). Thus, repair and protective mechanisms based on organic coatings might have a strong impact on the energy budget of mussels with corroded inner shell surfaces. Whether inner shell repair and maintenance is provided by the mantle tissue, or whether circulating hemocytes are involved in material deposition at sites of repair is also unclear at present [51]–[54]. We have not found indications (on a stereomicroscopic level, i.e 40× magnification) for internal shell corrosion in our previous experiments at higher temperatures and high densities of food algae [10], [15], although thickness of aragonite platelets in shell cross - sections was reduced in the summer experiment [15].

While external shell dissolution in high *p*CO_2_ waters has been described for several marine ectothermic animals with heavily calcified exoskeletons e.g. [12]–[15], [55], internal shell dissolution has, to our knowledge, only been documented in one other study: Clark et al. [56], using SEM, reported pitting of the spicules of echinoplutei when raised at a *p*CO_2_ of ca. 130 Pa. The larval skeleton of echinoid larvae is secreted by a primary mesenchyme cell syncitium and is thought to be in contact with the surrounding extracellular (coelomic) fluid [57], [58]. Assuming that pluteus larvae are as weak acid-base regulators as adult echinoids studied so far [59] it is likely that larval spicules are also in contact with extracellular fluids highly undersaturated with regard to calcium carbonate. It is unknown, whether spicule corrosion in pluteus larvae observed by Clark et al. [56] was related to food supply. This option should be investigated in future studies on echinoderm early life stages.

### Conclusion

We conclude that internal shell dissolution in the bivalve *M. edulis* from the Baltic Sea is tightly coupled to the energy budget: effects of food algae density in the treatment aquaria have a strong impact on inner shell integrity. These results demonstrate that maintenance of an intact nacre surface in an extrapallial fluid that is corrosive (even under control conditions, see [15]) requires energy input. We hypothesize that during strong *p*CO_2_ stress coupled to food limitation typical for the winter months, *M. edulis* might allocate resources towards conservation of somatic mass, thereby partially sacrificing nacre, thus utilizing an evolutionarily conserved plastic mechanism originating from the extreme requirements of life in an intertidal habitat. Our results also indicate that it is extremely difficult to infer rates of shell dissolution from empty shells subjected to corrosive seawater (e.g. [60]) to constrain calcification budgets (i.e. net vs. gross calcification), as there is great biological control over inner shell surface integrity. Elucidation of the fraction of the energy budget that is devoted to inner shell maintenance mechanisms under adverse carbonate system speciation states awaits investigation.

## References

[1] Doney SC, Balch WM, Fabry VJ, Feely RA (2009). Ocean acidification: A critical emerging problem for the ocean sciences.. Oceanography.

[2] Kroeker KJ, Kordas RL, Crim RN, Singh GG (2010). Meta-analysis reveals negative yet variable effects of ocean acidification on marine organisms.. Ecol Lett.

[3] Ries JB, Cohen AL, McCorkle DC (2009). Marine calcifiers exhibit mixed responses to CO_2_ induced ocean acidification.. Geology.

[4] Fabry VJ, Seibel BA, Feely RA, Orr JC (2008). Impacts of ocean acidification on marine fauna and ecosystem processes.. ICES J Mar Sci.

[5] Beniash E, Ivanina A, Lieb NS, Kurochkin I, Sokolova IM (2010). Elevated level of carbon dioxide affects metabolism and shell formation in oysters *Crassostrea virginica*.. Mar Ecol Prog Ser.

[6] Gaylord B, Hill TM, Sanford E, Lenz EA, Jacobs LA (2011). Functional impacts of ocean acidification in an ecologically critical foundation species.. J Exp Biol.

[7] Gazeau F, Gattuso JP, Dawber C, Pronker AE, Peene F (2010). Effect of ocean acidification on the early life stages of the blue mussel *Mytilus edulis*.. Biogeosciences.

[8] Kurihara H, Asai T, Kato S, Ishimatsu A (2008). Effects of elevated *p*CO_2_ on early development in the mussel *Mytilus galloprovincialis*.. Aquat Biol.

[9] Talmage SC, Gobler CJ (2010). Effects of past, present, and future ocean carbon dioxide concentrations on the growth and survival of larval shellfish.. Proc Natl Acad Sci USA.

[10] Thomsen J, Melzner F (2010). Seawater acidification does not elicit metabolic depression in the blue mussel *Mytilus edulis*.. Mar Biol.

[11] Lannig G, Eilers S, Pörtner HO, Sokolova IM, Bock C (2010). Impact of ocean acidification on energy metabolism of oyster, *Crassostrea gigas* – Changes in metabolic pathways and thermal response.. Mar Drugs.

[12] Hall-Spencer JM, Rodolfo-Metalpa R, Martin S, Ransome E, Fine M (2008). Volcanic carbon dioxide vents show ecosystem effects of ocean acidification.. Nature.

[13] Marshall DJ, Santos JH, Leung KMY, Chak WH (2008). Correlations between gastropod shell dissolution and water chemical properties in a tropical estuary.. Mar Environ Res.

[14] Lischka S, Buedenbender J, Boxhammer T, Riebesell U (2011). Impact of ocean acidification and elevated temperatures on early juveniles of the polar shelled pteropod *Limacina helicina*: mortality, shell degradation, and shell growth.. Biogeosciences.

[15] Thomsen J, Gutowska MA, Saphörster J, Heinemann A, Fietzke J (2010). Calcifying invertebrates succeed in a naturally CO_2_ - rich coastal habitat but are threatened by high levels of future acidification.. Biogeosciences.

[16] Tunnicliffe V, Davies KTA, Butterfield DA, Embley RW, Rose JM (2009). Survival of mussels in extremely acidic waters on a submarine volcano.. Nat Geosci.

[17] Addadi L, Joester D, Nudelman F, Weiner S (2006). Mollusk shell formation: a source of new concepts for understanding biomineralization processes.. Chem Eur J.

[18] Nudelman F, Shimoni E, Klein E, Rousseau M, Bourrat X (2008). Environmental- and cryo-scanning electron microscopy study of the forming nacreous layer from the shells of the bivalves *Atrina rigida* and *Pinctada margaritifera*.. J Struct Biol.

[19] Rousseau M, Lopez E, Coute A, Mascarel G, Smith DC (2005). Sheet nacre growth mechanism: a Voronoi model.. J Struct Biol.

[20] Cao L, Caldeira K (2008). Atmospheric CO_2_ stabilization and ocean acidification.. Geophys Res Lett.

[21] Lehmann A, Krauss W, Hinrichsen HH (2002). Effects of remote and local atmospheric forcing on circulation and upwelling in the Baltic Sea.. Tellus.

[22] Melzner F, Gutowska MA, Langenbuch M, Dupont S, Lucassen M (2009). Physiological basis for high CO_2_ tolerance in marine ectothermic animals: pre-adaptation through lifestyle and ontogeny?. Biogeosciences.

[23] Suzuki M, Saruwatari K, Kogure T, Yamamoto Y, Nishimura T (2009). An acidic matrix protein, Pif, is a key macromolecule for nacre formation.. Science.

[24] Weiss IM (2010). Jewels in the pearl.. ChemBioChem.

[25] Crenshaw MA, Neff JM (1969). Decalcification at the mantle-shell interface in molluscs.. Amer Zool.

[26] Burnett LE (1988). Physiological responses to air exposure: acid-base balance and the role of branchial water stores.. Amer Zool.

[27] Waite JH, Hochachka PW (1983). Quinone-tanned scleroproteins..

[28] Gutowska MA, Melzner F, Pörtner HO, Meier S (2010). Cuttlebone calcification increases during exposure to elevated seawater *p*CO_2_ in the cephalopod *Sepia officinalis*.. Mar Biol.

[29] Gutowska MA, Melzner F, Langenbuch M, Bock C, Claireaux G (2010). Acid-base regulatory capacity in the cephalopod *Sepia officinalis* exposed to environmental hypercapnia.. J Comp Physiol B.

[30] Checkley DM, Dickson AD, Takahashi M, Radich JA, Eisenkolb N (2009). Elevated CO_2_ enhances otolith growth in young fish.. Science.

[31] Wood HL, Spicer JI, Widdicombe S (2008). Ocean acidification may increase calcification rates, but at a cost.. Proc R Soc B Biol.

[32] Stumpp M, Wren J, Melzner F, Thorndyke MC, Dupont S (2011). CO_2_ induced acidification impacts sea urchin larval development I: elevated metabolic rates decrease scope for growth and induce developmental delay.. Comp Biochem Physiol A.

[33] Stuckas H, Stoof K, Quesada H, Tiedemann R (2009).

[34] Lewis E, Wallace DWR (1998). Program developed for CO_2_ system calculations..

[35] Roy RN, Roy LN, Vogel KM, Porter-Moore C, Pearson T (1993). The dissociation constants of carbonic acid in seawater at salinities 5 to 45 and temperatures 0 to 45°C.. Mar Chem.

[36] El-Dessouky HT, Ettouney HM (2002). Fundamentals of saltwater desalination.

[37] Riisgård HU, Møhlenberg F (1979). An improved automatic recording apparatus for determining the filtration rate of *Mytilus edulis* as a function of size and algal concentration.. Mar Biol.

[38] Riisgård HU, Larsen PS (2007). Viscosity of seawater controls beat frequency of water-pumping cilia and filtration rate of mussels *Mytilus edulis*.. Mar Ecol Prog Ser.

[39] Braby CE, Somero GN (2006). Following the heart: temperature and salinity effects on heart rate in native and invasive species of blue mussels (genus *Mytilus*).. J Exp Biol.

[40] Møhlenberg F, Riisgård HU (1978). Efficiency of particle retention in 13 species of suspension feeding bivalves.. Ophelia.

[41] Lafarga de la Cruz F, Valenzuela Espinoza E, Millan Nunez R, Trees CC, Santamaria del Angel E (2006). Nutrient uptake, chlorophyll *a* and carbon fixation by *Rodomonas* sp. (Cryptophyceae) cultured at different irradiance and nutrient concentrations.. Aquacult Eng.

[42] Gocke K, Rheinheimer G (1991). Influence of eutrophication on bacteria in two fjords of the Western Baltic.. Int Rev Ges Hydrobiol.

[43] Berge JA, Bjerkeng B, Pettersen O, Schaanning MT, Oxnevad S (2006). Effects of increased sea water concentrations of CO_2_ on growth of the bivalve *Mytilus edulis L*.. Chemosphere.

[44] Michaelidis B, Ouzounis C, Paleras A, Pörtner HO (2005). Effects of long-term moderate hypercapnia on acid-base balance and growth rate in marine mussels *Mytilus galloprovincialis*.. Mar Ecol-Prog Ser.

[45] Dugal LP (1939). The use of calcareous shell to buffer the product of anaerobic glycolysis in *Venus mercenaria*.. J Cell Comp Physiol.

[46] Akberali HB (1980). ^45^Calcium uptake and dissolution in the shell of *Scrobicularia plana* (da Costa).. J Exp Mar Biol Ecol.

[47] Akberali HB, Brear K, Currey JD (1983). Mechanical and morphological properties of the shell of *Scrobicularia plana* (da Costa) under normal and stress conditions.. J Mollus Stud.

[48] Jokumsen A, Fyhn HJ (1982). The influence of aerial exposure upon respiratory and osmotic properties of haemolymph from two intertidal mussels, *Mytilus edulis L.* and *Modiolus modiolus L*.. J Exp Mar Biol Ecol.

[49] Lindinger MI, Lauren DJ, McDonald DG (1984). Acid-base-balance in the sea mussel, *Mytilus edulis*. 3. Effects of environmental hypercapnia on intracellular and extracellular acid-base-balance.. Mar Biol Lett.

[50] Palmer AR (1992). Calcification in marine molluscs: How costly is it?. Proc Natl Acad Sci USA.

[51] Neff JM (1972). Ultrastructure of the outer epithelium of the mantle in the clam *Mercenaria mercenaria* in relation to calcification of the shell.. Tissue Cell.

[52] Mount AS, Wheeler AP, Paradkar RP, Snider D (2004). Hemocyte-mediated shell mineralization in the eastern oyster.. Science.

[53] Kadar E (2008). Haemocyte response associated with induction of shell regeneration in the deep-sea vent mussel *Bathymodiolus azoricus* (Bivalvia: Mytilidae).. J Exp Mar Biol Ecol.

[54] Kadar E, Lobo-da-Cunha A, Azevedo C (2009). Mantel to shell CaCO_3_ transfer during shell repair at different hydrostatic pressures in the deep-sea vent mussel *Bathymodiolus azoricus* (Bivalvia:Mytilidae).. Mar Biol.

[55] McClintock JB, Angus RA, Mcdonald MR, Amsler CD, Catledge SA (2009). Rapid dissolution of shells of weakly calcified Antarctic benthic macroorganisms indicates high vulnerability to ocean acidification.. Antarct Sci.

[56] Clark D, Lamare M, Barker M (2009). Response of sea urchin pluteus larvae (Echinodermata: Echinoidea) to reduced seawater pH: a comparison among tropical, temperate, and a polar species.. Mar Biol.

[57] Decker GL, Morrill JB, Lennarz WJ (1987). Characterization of sea urchin primary mesenchyme cells and spicules during biomineralization *in vitro*.. Development.

[58] Wilt FH, Killian C, Hamilton P, Croker L (2008). The dynamics of secretion during sea urchin embryonic skeleton formation.. Exp Cell Res.

[59] Miles H, Widdicombe S, Spicer JI, Hall-Spencer J (2007). Effects of anthropogenic seawater acidification on acid-base balance in the sea urchin *Psammechinus miliaris*.. Mar Poll Bull.

[60] Nienhuis S, Palmer AR, Harley CDG (2010). Elevated CO_2_ affects shell dissolution rate but not calcification rate in a marine snail.. Proc R Soc B.

